# High Efficient Reduction of Graphene Oxide via Nascent Hydrogen at Room Temperature

**DOI:** 10.3390/ma11030340

**Published:** 2018-02-27

**Authors:** Qiqi Zhuo, Jijun Tang, Jun Sun, Chao Yan

**Affiliations:** 1 College of Material Science & Engineering, Jiangsu University of Science and Technology, 2 Meng-Xi Road, Zhenjiang 212003, China; zqq88263268@just.edu.cn (Q.Z.); tangjijunnju@126.com (J.T.); 2 College of Chemistry, Chemical Engineering and Material Science, Soochow University, 199 Ren-Ai Road, Suzhou 215123, China ; sunjun@suda.edu.cn

**Keywords:** graphene oxide, graphene, reductants

## Abstract

To develop a green and efficient method to synthesize graphene in relative milder conditions is prerequisite for graphene applications. A chemical reducing method has been developed to high efficiently reduce graphene oxide (GO) using Fe_2_O_3_ and NH_3_BH_3_ as catalyst and reductants, respectively. During the process, environmental and strong reductive nascent hydrogen were generated surrounding the surface of GO sheets by catalyst hydrolysis reaction of NH_3_BH_3_ and were used for reduction of GO. The reduction process was studied by ultraviolet absorption spectroscopy, Raman spectroscopy, and Fourier transform infrared spectrum. The structure and morphology of the reduced GO were characterized with scanning electron microscopy and transmission electron microscopy. Compared to metal (Mg/Fe/Zn/Al) particles and acid system which also use nascent hydrogen to reduce GO, this method exhibited higher reduction efficiency (43.6%). Also the reduction was carried out at room temperature condition, which is environmentally friendly. As a supercapacitor electrode, the reversible capacity of reduced graphene oxide was 113.8 F g^−1^ at 1 A g^−1^ and the capacitance retention still remained at 90% after 200 cycles. This approach provides a new method to reduce GO with high reduction efficiency by green reductant.

## 1. Introduction

Graphene has gained large attention because of its excellent mechanical, electrical, thermal and optical properties. Special electronic structure and outstanding properties provide graphene with great potential in the applications of sensors [1,2,3], electronics [4,5,6], batteries [7,8], and nanocomposites [9,10,11]. Thus, a facile synthesis of graphene with large quantities is pursued. To date, many techniques have been introduced to synthesize graphene, such as chemical reduction of GO [12,13,14,15], liquid-phase exfoliation [16,17], micro-mechanical exfoliation [18], chemical vapor deposition [19,20,21], etc. Overall, the chemical reduction of GO is believed to be one of the most promising methods to synthesize reduced graphene oxide (r-GO) with low cost and large-scale productivity [14]. Until now, the graphene oxide can be large scale prepared by exfoliation of graphite oxide, which is obtained by oxidizing graphite using oxidants and strong acid [22,23]. GO nanosheets have a lot of functional groups—including epoxide, carbonyl, hydroxyl, and carboxy groups—which make it hydrophilic [24,25]. Therefore, GO sheets can be dispersed uniformly in water and many kinds of organic solvent due to electrostatic repulsion of carboxylate groups on GO sheets that with a negative charge [14]. However, metal ions can neutralize the charges on the GO sheets and destabilize the resulting dispersions [26]. For instance, NaCl would make hydrophilic GO aggregate in water [27]. 

Until now, various inorganic and organic reductants have been exploited for chemical reduction of GO [13,14]. For instance, hydrazine and its derivatives [24] are effective and efficient reductants for GO reduction [14]. However, these reductants are highly toxic to both living organisms and the environment which limited their usage [28]. Meanwhile, some relatively low-toxicity reductants like hydroxylamine [29], NaBH_4_ [12,30], urea [31], sugar [32], L-ascorbic acid [33], and sodium citrate [34] are developed for reduction of GO. However, most of these reductants should be employed at a relatively high temperature beyond 90 °C, which will increase the defects in r-GO sheets as examined by Raman spectroscopy and X-ray photoelectron spectroscopy [33]. It is worthwhile to exploit a green and efficient method to prepare graphene from reducing of GO in relative milder conditions. Recently, many studies have focused on chemically reducing GO by green reductants at room temperature. As a strong reducing and environmentally benign agent [14], nascent hydrogen can be used for efficiently reducing of GO. Usually, nascent hydrogen was generated by reaction of acid solution with metal powders. Until now, GO has been reported to be reduced by nascent hydrogen generated by metals of different electrochemical potential (−0.44 V for Fe [35], −0.76 V for Zn [36], −1.66 V for Al [37], −2.37 V for Mg [38]) However, hydrophobic metal powders and hydrophilic GO cannot contact well. A great amount of nascent hydrogen generated around metal powders surface would quickly form non-reducing hydrogen instead to reduce GO, which results in low reduction efficiency.

Ammonia borane (NH_3_BH_3_) is a stable material for chemically storing hydrogen and an excellent reducing agent [14]. Nascent hydrogen can be generated by release of hydrogen atoms from NH_3_BH_3_ through methanolysis [39,40] or hydrolysis [41,42] in solution. Compared to the thermal dehydrogenation process which requires high temperature, NH_3_BH_3_ can release hydrogen at room temperature by hydrolysis reaction with the presence of noble or non-noble metal catalysts (Co. [43], Ru [44], Pd [45], etc.). In our previous work, Co_3_O_4_ was used as catalyst for high efficient reduction of GO by the hydrolysis of NH_3_BH_3_. The catalytic rate for NH_3_BH_3_ hydrolysis of Co_3_O_4_ is as high as 3~5 times of Fe_2_O_3_ and about 10~20 times of CuO/Cu_2_O [46]. However, the high reaction rate would generate large quantity of nascent hydrogen too fast, thus harming the formation of porous graphene and reducing the capacitance of graphene nanomaterial, which limits its application in the fields of supercapacity. Also, the large amount hydrogen produced in a short time may present some security risks.

Based on the salting out effect of GO, this work describe a mild method for the reduction of GO using nascent hydrogen generated by NH_3_BH_3_ and Fe_2_O_3_ as the reducing agent and catalyst, respectively. UV-vis absorption spectroscopy, Raman spectroscopy, and Fourier transform infrared spectrum were used to study the reducing process. The structure and morphology of the reduced graphene oxide were characterized by scanning electron microscopy (SEM) and transmission electron microscopy (TEM). This method showed higher nascent hydrogen reduction efficiency and relatively lower defects than metal/acid reduction system. Besides, supercapacitor with the prepared r-GO materials as electrode was fabricated to study their electrochemical properties.

## 2. Materials and Methods

### 2.1. Materials

NH_3_BH_3_, FeCl_3_ and HCl were purchased from Sinopharm Chemical Reagent, Shanghai, China. GO was prepared by the modified Hummers method [22]. 

### 2.2. Reduction of GO

Firstly, as-prepared GO was dispersed in deionized water by ultrasonic treatment for 30 min to form a homogeneous aqueous solution (0.1 mg/mL). 1 mL of 0.1 M FeCl_3_ and 10 mg NH_3_BH_3_ were added to the 10 mL GO (1 mg) solution in sequence. After a 60 min reaction, a black suspension solution was obtained. Finally, the product was followed by washing with dilute HCl and deionized water five times. 

### 2.3. Characterization

Raman spectra were carried out using a Renishaw Micro-Raman spectrometer System, UK with an excitation source of 532 nm wavelength incident laser. UV-vis absorption spectroscopy detection was performed by a LAMBDA 750 spectrometer (PerkinElmer, Waltham, MA, USA). The transmission electron microscopy (TEM, FEI, Hillsboro, OR, USA) was used to investigate surface morphology of r-GO/Fe_2_O_3_ and r-GO with an accelerating voltage of 200 kV. Fourier transform infrared (FTIR) spectra of the samples were carried with HYPERION 2000 spectrometer (Bruker, karlsruhe, Germany) in the range of 600–4000 cm^−1^. 

### 2.4. Electrochemical Measurements

The electrochemical performances of r-GO and GO were evaluated using a three-electrode setup in 2 M KOH solution. Pt foil, Hg/HgO, and the as-prepared materials were used respectively as counter electrode, reference electrode, and working electrode. The working electrode was prepared by coating a paste onto nickel foam, using r-GO as active material, poly(tetrafluoroethylene) as binder, and acetylene black as conductive additive with a weight ratio of 80: 10: 10 in NMP. Finally, the nickel foam was dried at 80 °C and the weight of active material on each electrode was about 1.5 mg.

## 3. Results and Discussion

### 3.1. Schematic of Reduction Process

**Figure 1:**
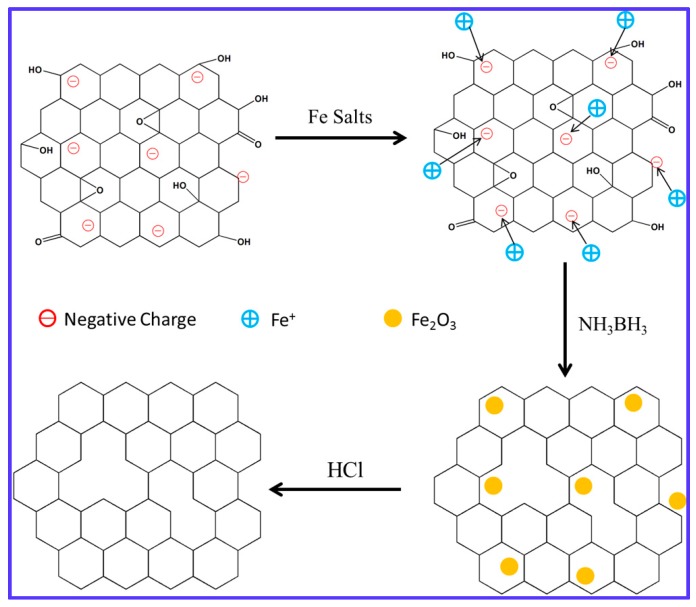
Schematic of reducing GO sheets.

Figure 1 shows the schematic of the reducing GO. Due to ionization of the carboxylic acid and phenolic hydroxyl groups on the GO sheets, GO sheets shows highly negative charge when dispersed in water. FeCl_3_ solution would neutralize the negative charge of GO after adding to the GO solution, thus make GO sheets agglomerate. Then NH_3_BH_3_ was added and a black suspension of r-GO/Fe_2_O_3_ was obtained. Fe_2_O_3_ NPs could catalyze the hydrolysis reaction of NH_3_BH_3_ to generate nascent hydrogen to reduce GO nanosheets. At last, Fe_2_O_3_ nanoparticles were removed away by HCl and the pure r-GO was collected.

### 3.2. Structural and Morphological Investigations

**Figure 2:**
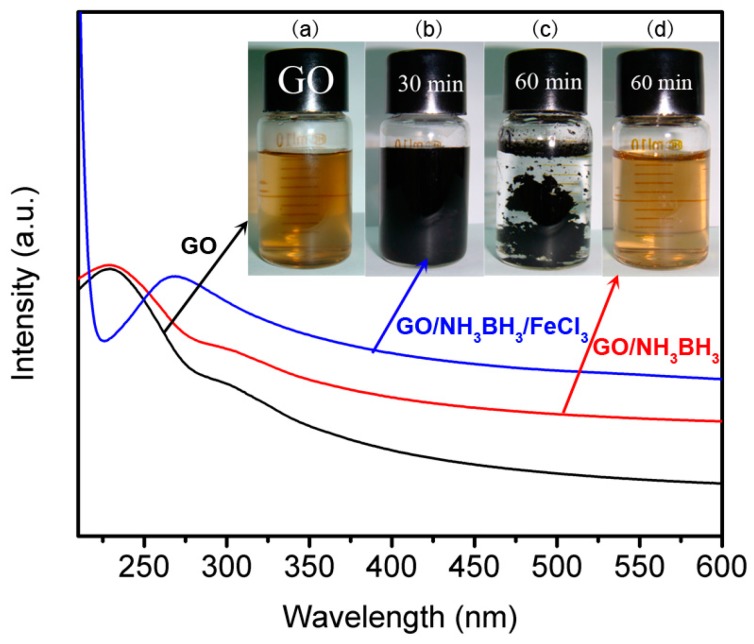
Ultraviolet visible spectroscopy of GO (**a**) and GO/NH_3_BH_3_ with (**b** for 30 min, **c** for 60 min) or without (**d**) FeCl_3_ during the reduction process. Inset picture is the corresponding photos of the products.

Ultraviolet visible spectroscopy was used to study the reduction process. The ultraviolet visible absorption peak of GO/FeCl_3_ solution shifted from 232 nm (Figure 2a) to 268 nm (Figure 2b) after 30 min, and the solution color changed from brown to black, indicating that the highly conjugated structure like that of graphite was formed gradually and GO was reduced to graphene [12,32,35]. Compared to the system of GO/FeCl_3_, the reduction rate of pure GO without FeCl_3_ was much slower. Meanwhile, the solution color did not have obvious change with the reaction time extended even to 60 min (Figure 2d). This result demonstrated that iron salts can work as the catalyst to accelerate the hydrolysis reaction of NH_3_BH_3_ greatly to generate nascent hydrogen and reduce GO. Below showed the reduction process of GO:

(1) $${\mathrm{NH}}_{3}{\mathrm{BH}}_{3}+2H_{2}O\overset{{\mathrm{Fe}}_{2}O_{3}}{\to }{\;\mathrm{NH}}_{4}^{+}+{\mathrm{BO}}_{2}^{-}+6H$$

(2) $$\mathrm{GO}+\mathrm{aH}\to r-\mathrm{GO}+{\mathrm{bH}}_{2}O+{\mathrm{cH}}_{2}$$

In the first step, Fe_2_O_3_ NPs were absorbed on the surface of GO and then a mass quantity of nascent hydrogen formed around the Fe_2_O_3_ NPs by the hydrolysis of NH_3_BH_3_, which could be directly used to reduce GO. In the second step, GO was reduced by nascent hydrogen.

**Figure 3:**
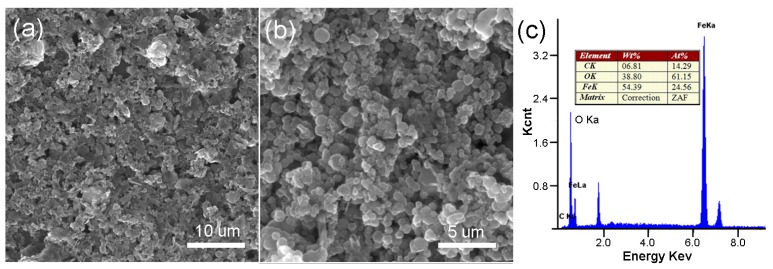
SEM (scanning electron microscopy) images of the r-GO/Fe_2_O_3_ NPs with different magnifications (**a**,**b**) and corresponding EDX (**c**).

**Figure 4:**
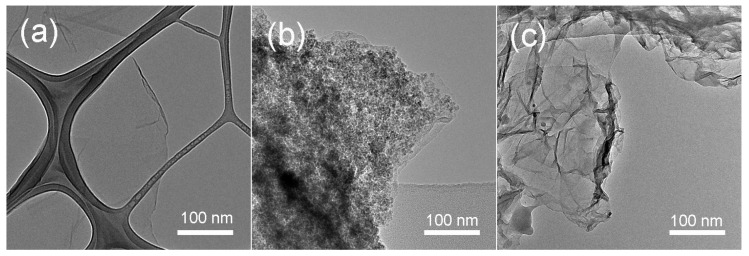
TEM (transmission electron microscopy) images of the (**a**) GO, (**b**) r-GO/Fe_2_O_3_, and (**c**) r-GO sheets.

Figure 3 shows the SEM image of r-GO/Fe_2_O_3_ NPs with different magnifications. There are numerous Fe_2_O_3_ NPs decorated on r-GO sheets. Corresponding EDX result indicated the existence of Fe, O, and C elements. The morphology was also characterized by TEM as shown in Figure 4. It is clearly seen that the Fe_2_O_3_ NPs disperse uniformly on the r-GO sheets (Figure 4b). No nanoparticles can be seen on pure GO sheets (Figure 4a). After being washed with HCl, Fe_2_O_3_ NPs were removed completely and pure r-GO was obtained (Figure 4c).

**Figure 5:**
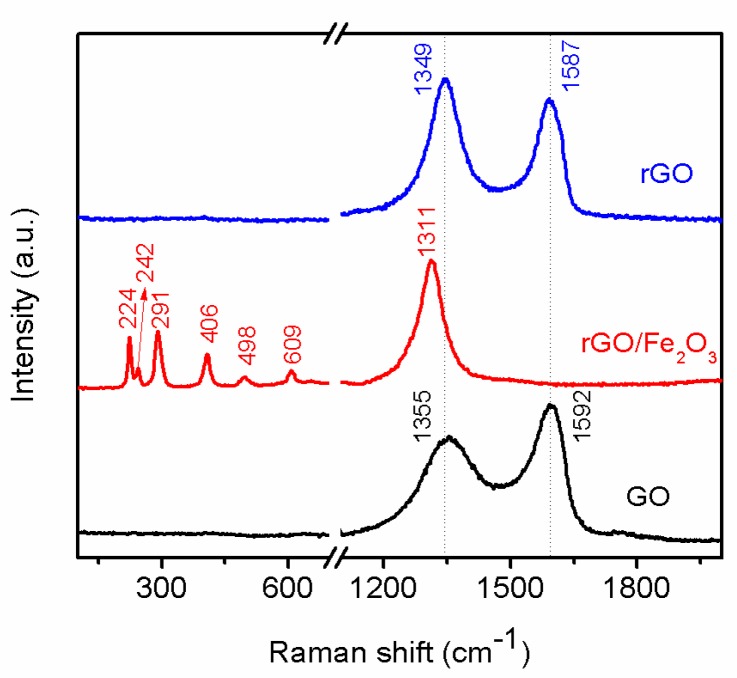
Raman spectra of GO, r-GO/Fe_2_O_3_, and r-GO.

Raman scattering is an important, non-destructive tool to characterize the change in the molecular structural of carbon based materials. The reducing process of GO was also investigated by Raman spectroscopy. The Raman spectrum (Figure 5) of GO showed two prominent peaks at 1355 and 1592 cm^−1^, corresponding to the D band and G band, and the intensity ratio of D band to G band (I_D_/I_G_) which indicates the degree of the disorder such as defects, ripples, and edges [14] is approximately 0.64. The red curve is the Raman spectrum of r-GO/Fe_2_O_3_. Many peaks can be observed, which are located at 224, 242, 291, 406, 498, 609, and 1311 cm^−1^, respectively [47]. All these peaks correspond to α-Fe_2_O_3_ phase. The Raman peaks appearing at 224 and 498 cm^−1^ are assigned to A_1g_ mode, and peaks at 291, 406, and 609 cm^−1^ are assigned to E_g_ modes. Meanwhile the peak observed at 1311 cm^−1^ is assigned to hematite two-magnon scattering. It is difficult to identify the carbon peak which should be located at ~1350 and ~1590 cm^−1^ because it has a strong fluorescent scattering of α-Fe_2_O_3_. After washed by HCl, other peaks between 200~1320 totally disappeared, showing that Fe_2_O_3_ NPs have been removed. On the contrary, the peaks of D and G band of carbon appeared and the I_D_/I_G_ ratio of r-GO gradually increased to 1.19, which matches well with the results reported in the previous reports [12,14], indicating that GO has been reduced to r-GO completely.

**Figure 6:**
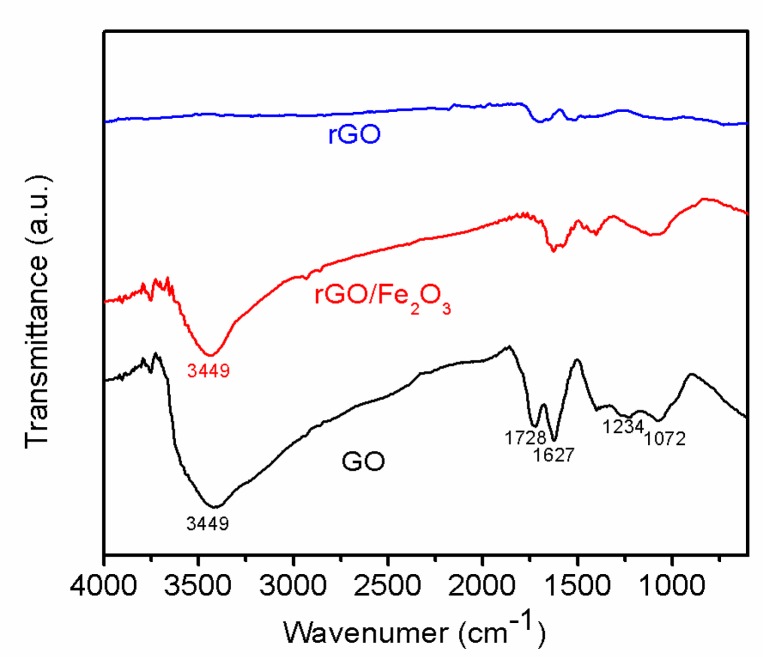
FTIR spectra of GO, r-GO/ Fe_2_O_3_, and r-GO.

The reduction process can also be detected by FTIR. The FTIR spectra (Figure 6) of GO showed peaks at 1072/1234 cm^−1^, 1728 cm^−1^, and 3449 cm^−1^ corresponding to the C–O, C=O, and –OH, which indicates there are lots of oxygenous groups on GO sheets. The FTIR spectra of r-GO showed the intensity of all peaks corresponding to the oxygenous groups decrease dramatically compared to that of primal GO, which further indicate GO nanosheets have been reduced to r-GO nanosheets [12]. 

### 3.3. Measure Nascent Hydrogen Reduction Efficiency

**Figure 7:**
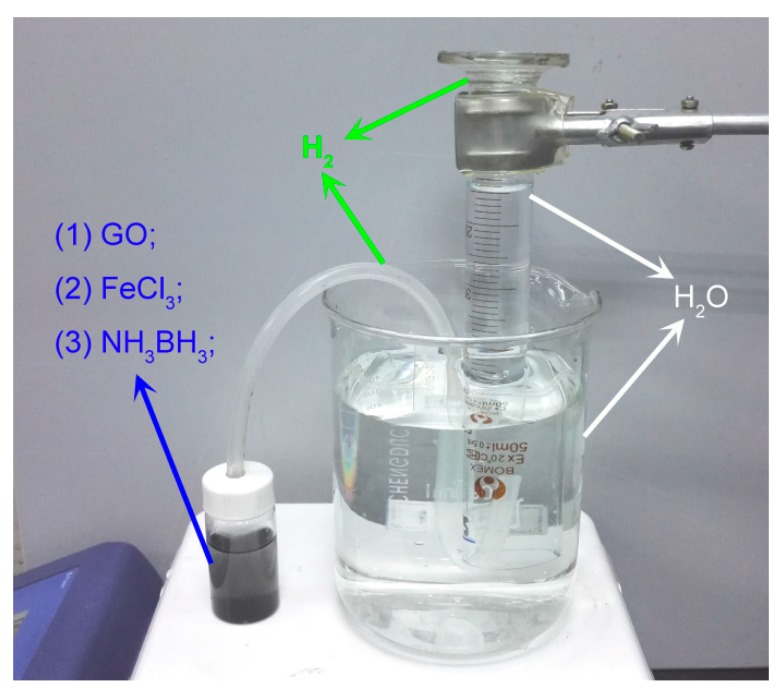
The instrument to measure the H_2_ contends.

During the reduction process, many H atoms generated surrounding Fe_2_O_3_ NPs would quickly form H_2_. By contrast, H_2_ has no reducibility at room temperature [14]. Then a simple instrument was designed to collect H_2_ produced and measure the content so as to quantify the reduction efficiency, as Figure 7 shows. The reduction ratios (H %) was calculated to be 43.6 % according to following formula:

(3) $$H\;\%=1-\frac{V_{H_{2}-\mathrm{trapped}}}{V_{H_{2}-\mathrm{theoretical}}}$$

The reduction efficiency of different metal (Mg/Fe/Zn/Al) and acid reduction systems were also measured (Table 1). Compared to metal/acid reduction method, our method showed higher efficiency. It may be due to hydrophobic metal particles in the metal/acid system being unable to contact well with hydrophilic GO sheets. As a result, the nascent hydrogen which was formed around metal particles is tough to reduce GO sheets. In our method, much nascent hydrogen could be produced around the GO sheets surface as Fe_2_O_3_ NPs were in-site formed on the surface of GO sheets and could catalyze the hydrolysis of NH_3_BH_3_, thus GO was reduced more efficiently.

### 3.4. Electrochemical Properties of r-GO

**Figure 8:**
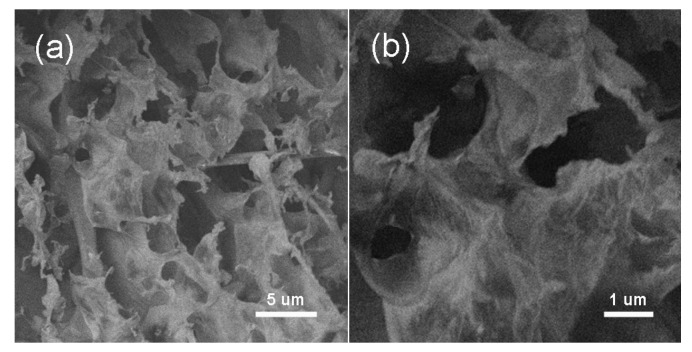
SEM images of the r-GO with different magnifications (**a**,**b**).

Due to the hydrophilic functional group is removed in the reduction process of GO, graphene nanosheets were easy to stack or aggregate so that the specific surface area was decreased. As a result, the capacitance of graphene nanosheets was reduced, which would limit its application in the fields of supercapacity. By our method, the gas generated in the reduction process could efficiently prevent nanosheets to aggregate and r-GO with porous structure was obtained (Figure 8). 

**Figure 9:**
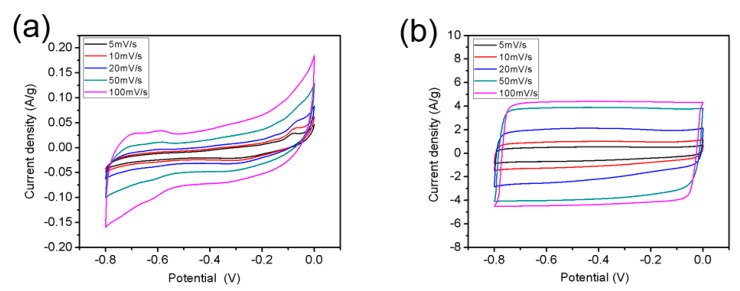
CV curves of GO (**a**) and r-GO (**b**) at various scan rates from 5 to 100 mV/s.

The electrochemical properties of r-GO were investigated by cyclic voltammetry (CV) test, in which 2 M KOH was worked as electrolyte. Figure 9 shows the CV curves of GO and r-GO at various scan rates of 5, 10, 20, 50, and 100 mV s^−1^. The rectangular shape of the CV curves belong to r-GO can be attributed to good capacitive performance of the carbon based materials, and the current density of r-GO was 20 times as high as that of GO at the scan rate of 100 mV s^−1^. 

**Figure 10:**
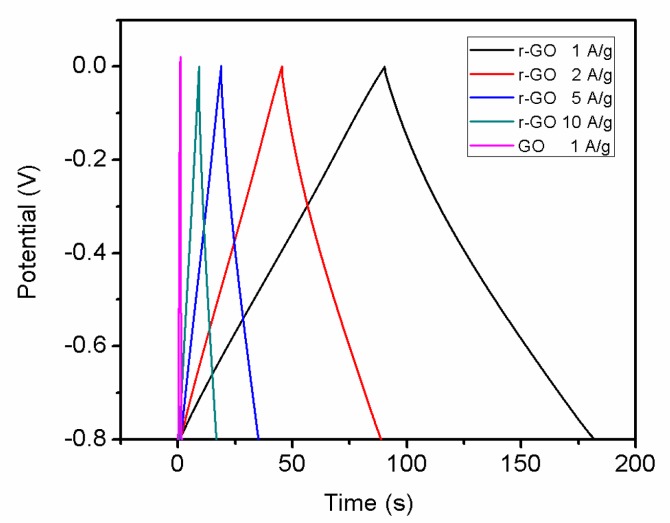
Galvanostatic charge–discharge curves of the symmetric supercapacitor at various current densities in 2 M KOH.

**Figure 11:**
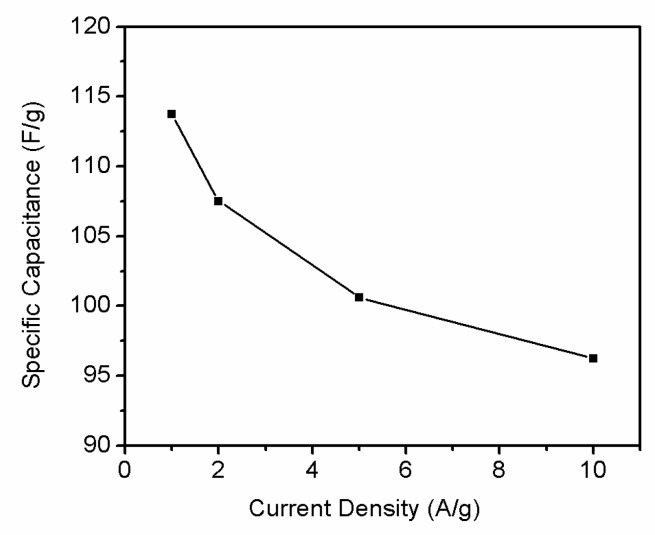
Specific capacitance of the symmetric supercapacitor at various current densities in 2 M KOH.

Rate capability test is important to evaluate the power performance of electrodes materials. Figure 10 shows the current galvanostatic discharge curves of r-GO electrode at different current densities. With the current density increase, shorter reaction time can be observed. Figure 11 shows the specific capacities of r-GO electrodes at current density of 1, 2, 5 and 10 A g^−1^, respectively. It can be seen with the increase of current density, the capacity decrease from 113.8 F g^−1^ at 1 A g^−1^ to 96.25 F g^−1^ at 10 A g^−1^. This can be attributed to the surface area of electrode contact with electrolyte reduced with the increase of current density. 

**Figure 12:**
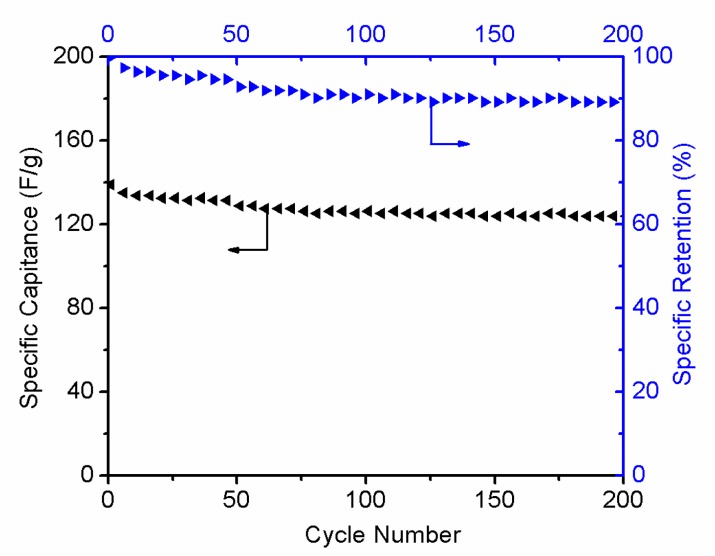
Cycling performance of r-GO electrodes at 1 A g^−1^ and specific retention.

Cycling stability is another important supercapacitor parameter. Figure 12 shows the cyclic performances and specific retention of the r-GO electrodes at 1 A g^−1^. After 200 cycles, the capacitance retention of the r-GO still remains at 90%, revealing that the electrodes exhibited good stability behaviors as supercapacitor electrode material.

## 4. Conclusions

Nascent hydrogen as an environmentally benign and strong reducing agent was used to efficiently reduction graphene oxide. In metal (Mg/Fe/Zn/Al) and acid reduction systems, hydrophobic metal powders and hydrophilic GO cannot contact well, which would result in low nascent hydrogen reduction ratio. In this article, a chemical method using Fe_2_O_3_ and NH_3_BH_3_ as catalyst and reductants was developed for high efficient reduction of GO. During the reduction process, Fe_2_O_3_ NPs were spontaneously formed on GO sheets surface due to the salting effect and catalyze the hydrolysis reaction of NH_3_BH_3_ to general nascent hydrogen at room temperature. The nascent hydrogen reduction ratio was up to 43.6%. Ultraviolet visible spectra, Fourier transform infrared spectrum, and Raman spectra demonstrated that almost all oxygenous groups on GO sheets were removed during the reduction process. As a supercapacitor electrode, the reversible capacity of reduced graphene oxide was 113.8 F g^−1^ at 1 A g^−1^ and the capacitance retention still remains at 90% after 200 cycles. Also the reaction was conducted at room temperature, which led to fewer defects during the reduction process. We expect that this method opens up a new way to reduce GO with high reduction efficiency and low defect under a mild condition.

## Figures and Tables

**Table 1:** Reduction efficiency of different metal/acid systems and our method.

Reduction Method	Raman (ID/IG)	H (%)	Reference
Mg/HCl	1.52	10.3	[38]
Fe/HCl	1.38	9.8	[35]
Zn/HCl	1.49	29.6	[36]
Al/HCl	1.32	7.2	[38]
NH_3_BH_3_/Fe_2_O_3_	1.19	43.6	this paper
